# Creating Gerontological Space for Generative AI in the Classroom

**DOI:** 10.1093/geroni/igaf122.1549

**Published:** 2025-12-31

**Authors:** Candace Brown, Meredith Troutman-Jordan

**Affiliations:** University of North Carolina, University of North Carolina, Charlotte, North Carolina, United States; University of North Carolina Charlotte, Charlotte, North Carolina, United States

## Abstract

The discussion of Generative AI (GAI) and its uses, purposes, and misuse have exponentially increased in the last several years, highlighting the need for instructors to learn more about GAI tools and how they can effectively navigate the GAI space in their classroom. The University of North Carolina, Charlotte supports ethical and responsible use of artificial intelligence to enhance academic teaching and learning of faculty and students who engage in this technology while responsibly sustaining high standards of integrity. This presentation will: 1) discuss challenges and opportunities from faculty and student perspectives of GAI; 2) exemplify GAI development and use in gerontology courses; 3) provide resources for attendees interested in developing GAI into their course curriculum; and, 4) challenge participants to consider how they may build faculty capacity for GAI integration in their own institutions.

